# Minimally Invasive Versus Open Parastomal Hernia Repair: A Comprehensive Systematic Review and Meta‐Analysis

**DOI:** 10.1002/wjs.70013

**Published:** 2025-07-25

**Authors:** Ahmed Abdelsamad, Mohammed Khaled Mohammed, Mohamed Badr Almoshantaf, Aya Alrawi, Ziad A. Fadl, Ziad Tarek, Nada Osama Aboelmajd, Torsten Herzog, Florian Gebauer, Nada K. Abdelsattar, Taha Abd‐Elsalam Ashraf Taha

**Affiliations:** ^1^ Department of Surgery II University of Witten — Herdecke Witten Germany; ^2^ Oncological Surgery Department Section Head of Robotic Surgery Knappschaft Vest‐ Hospital Recklinghausen Germany; ^3^ Faculty of Medicine Cairo University Al Giza Egypt; ^4^ Department of General Surgery Ealing Hospital LNWH Trust London UK; ^5^ Faculty of Medicine Fayoum University Fayoum Egypt; ^6^ Faculty of Medicine South Valley University Qena Egypt; ^7^ Department of Surgery Ruhr‐Bochum University Bochum Germany; ^8^ Oncological Surgery Department Helios University Hospital Wuppertal Germany

**Keywords:** meta‐analysis, minimal invasive surgery, open surgery, parastomal hernia, postoperative outcomes

## Abstract

**Background:**

Parastomal hernia is a frequent and complex complication following stoma creation, often requiring surgical intervention. The optimal repair technique remains a subject of debate, with open and minimally invasive approaches each presenting distinct advantages and limitations. This meta‐analysis systematically compares these techniques for parastomal hernia repair, evaluating key clinical outcomes including short‐ and long‐term results.

**Methods:**

We performed a comprehensive systematic review and meta‐analysis following PRISMA guidelines, searching the PubMed, Web of Science, Scopus, and Cochrane Library databases through October 2024. Studies were included if they involved adult patients undergoing parastomal hernia repair using open and minimally invasive techniques, and reported at least two relevant clinical outcomes. Data were analyzed using Review Manager 5.4. Risk ratios (RRs) and mean differences (MDs) were calculated, with heterogeneity assessed through *p*‐values. A random‐effects model was applied for analyses with substantial heterogeneity.

**Results:**

Minimally invasive approaches were associated with favorable outcomes compared to open surgery in several key parameters. Laparoscopic repair significantly reduced hospital stay by an average of 4 days compared to open techniques (*p* < 0.00001) and showed significantly lower complication rates, including surgical site infections (RR: 0.37– 0.63, *p*‐values 0.02–0.03). Mortality was also significantly reduced in the laparoscopic group (RR: 0.18; *p* = 0.0009) while recurrence rates showed no significant difference between approaches. Robotic‐assisted repair demonstrated a potential reduction in operative time (RR = 0.60, *p* = 0.007); however, this finding is based on a limited sample and should be interpreted with caution.

**Conclusion:**

Laparoscopic repair of parastomal hernias provides significant advantages over open surgery including reduced morbidity, shorter hospital stays, and fewer complications. Although limited by sample size, robotic‐assisted repair seems promising in reducing operative time and may offer added value in complex or recurrent cases; however, further research is needed to validate its cost‐effectiveness and long‐term outcomes. These findings support the preferential use of a minimally invasive approach in parastomal hernia repair whenever feasible while acknowledging that an individualized approach may still be necessary based on patient‐specific factors and institutional resources.

## Graphical abstract

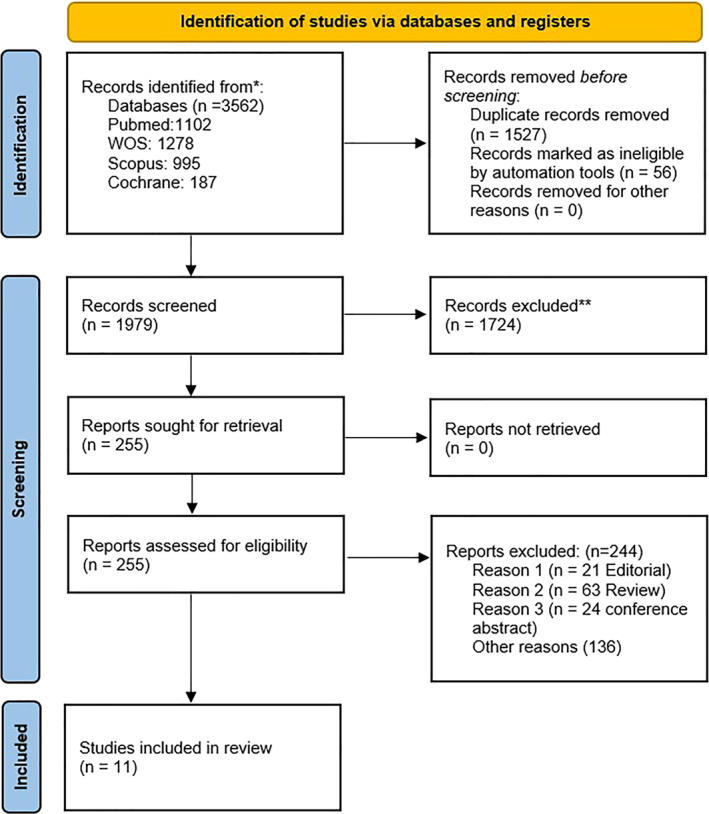

**Abbreviations**
MDs: mean differences
MIS: minimally invasive surgery
NOS: The Newcastle–Ottawa Scale
PRISMA: Preferred Reporting Items for Systematic Reviews and Meta‐analyses
ROB: Risk of Bias
RRs: Risk ratios
UTI: Urinary tract infection

## 1 Introduction

Parastomal hernia is one of the most common and challenging complications following stoma creation, often requiring surgical intervention [1, 2].

Over the years, several surgical techniques have been developed to repair parastomal hernias including open surgery, laparoscopic, and robotic‐assisted approaches. Each method offers unique advantages and challenges, but the debate about which approach yields the best clinical outcomes continues [3, 4, 5].

Recent advancements in minimally invasive techniques, such as laparoscopic and robotic‐assisted approaches, have shown potential in reducing complications, hospital stays, and recovery times compared to traditional open surgery [4, 6, 7, 8, 9]. Specifically, the laparoscopic approach has gained popularity due to its low cost and its minimally invasive nature, which often leads to reduced postoperative pain, shorter hospital stays, and faster recovery [8, 9, 10].

However, concerns remain regarding its effectiveness in complex cases and the learning curve associated with its application [11]. On the other hand, the robotic‐assisted approach, which provides enhanced precision and control, has also shown promise but may come with increased costs and longer operative times [9, 12, 13, 14].

This meta‐analysis aims to systematically compare the outcomes of open and minimally invasive approaches in parastomal hernia repair, focusing on parameters such as operative time, hospital stay, recurrence rates, postoperative complications, and overall patient outcomes. By synthesizing data from various studies, this review seeks to provide clarity on the most effective surgical approach for parastomal hernia repair, guiding clinical decision‐making in surgical practice.

## 2 Methods

**FIGURE 1:**
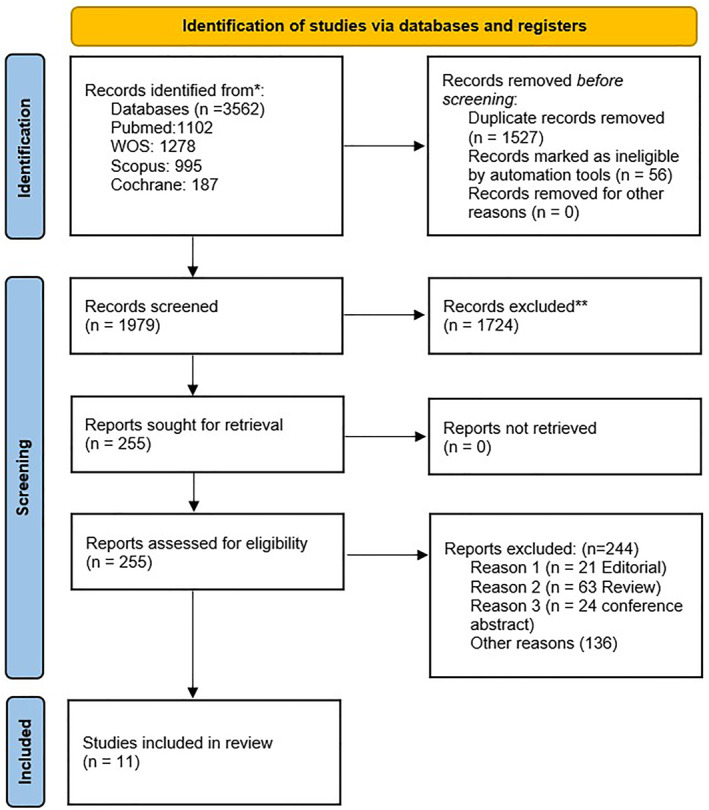
PRISMA flow diagram.

This systematic review and meta‐analysis were performed following the guidelines outlined in the preferred reporting items for systematic reviews and meta‐analysis (PRISMA) statement [15, 16]. Following the removal of duplicates, a total of 1979 records were screened by title and abstract, of which 255 full‐text articles were assessed for eligibility. Ultimately, 11 studies met the inclusion criteria and were included in the final analysis (Figure 1).

### 2.1 Eligibility Criteria

Studies were considered eligible if they met the following criteria: (1) encompassed adult patients (aged > 18 years) diagnosed with a parastomal hernia, irrespective of etiology or size; (2) focused on the laparoscopic repair of parastomal hernia, utilizing any described technique; (3) compared laparoscopic repair with open approaches; and (4) reported at least one relevant outcome. Studies were excluded if they were single‐arm studies, conference abstracts, case reports, case series, cross‐sectional, review articles, in vitro, or animal studies.

### 2.2 Search Strategy

**FIGURE 2:**
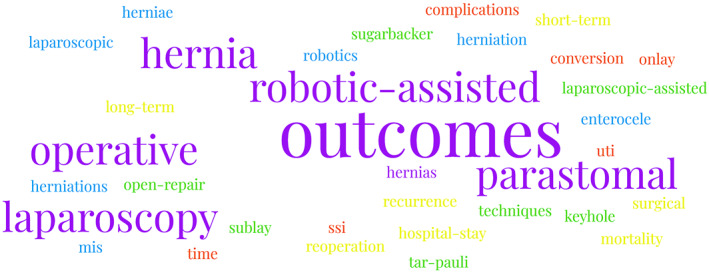
Word cloud.

A comprehensive search was executed across four databases: PubMed, Web of Science, Scopus, and Cochrane Library. The following search terms were used: (Parastomal) AND (hernia OR hernias OR Enterocele OR herniae OR herniations OR herniation). The search was conducted from database inception to October 5th, 2024. A visual representation of the search terms and their relevance is provided in Figure 2 as a word cloud, highlighting the focus areas of our systematic review, as per the Supporting Information S1: Appendix 1.

### 2.3 Study Selection

Retrieved studies were initially managed using EndNote X9 reference management software and then exported to Microsoft Excel for screening. A two‐stage screening protocol was implemented: (1) an initial screening of titles and abstracts, followed by (2) a full‐text review of articles that were deemed potentially eligible. Two independent reviewers performed each stage of the screening process. Discrepancies were resolved through discussion or, if necessary, consultation with a third, senior reviewer.

After applying these criteria, 11 studies [17, 18, 19, 20, 21, 22, 23, 24, 25, 26, 27] were identified as eligible for inclusion. Data were extracted from these studies, encompassing a total of 2834 patients, as detailed in Table 1 [17, 18, 19, 20, 21, 22, 23, 24, 25, 26, 27].

**TABLE 1:** Studies characteristics.

	Author and year	Country	Study design	F. up (months)	Surgical approach	Surgical technique	Number of patients
1	DeAsis et al. [17]	US	Retrospective cohort review	17	Laparoscopic	Sugar‐baker technique	25
					Laparoscopic	Keyhole	18
					Open		6
2	Dewulf et al. [18]	Europe	Observational design	12	Laparoscopy	Modified sugar‐baker	5
					Robotic‐assisted	Modified sugar‐baker, keyhole, and TAR Pauli	10
3	Halabi et al. [19]	US	A retrospective review	54	Laparoscopy		222
					Open		1945
4	Helgstrand et al. [20]	Denmark	Retrospective analysis	20	Laparoscopy	Keyhole and sugar‐baker, 5 conversion	118
					Open	On‐lay, sublay, keyhole, and sugar‐baker	56
5	Javaid et al. [21]	UK	Retrospective analysis	17	Laparoscopy		29
					Open		18
6	Keller et al. [22]	USA	Retrospective cohort	42	Open	Sublay, onlay, intraperitoneal, or 1ry suture repair.	31
				12	Laparoscopy	Sugar‐baker	31
7	Kohler et al. [23]	Austria	Retrospective cohort	54	Open		63
					Laparoscopic		72
8	Lambrecht [24]	Norway	Prospective observational	12	Laparoscopic	Modified sugar‐baker	6
					Robotic‐assisted		9
9	McLemore et al. [25]	USA	Retrospective cohort	65	Open		30
				20	Laparoscopic		19
10	Reali et al. [26]	UK	Prospective observational	285	Open		41
					Laparoscopic	Sugar‐baker	18
11	Ripoche et al. [27]	France	Retrospective cohort	126	Midline laparotomy		32
					Local approach		29
					Laparoscopic		10

### 2.4 Data Extraction

Two reviewers independently extracted data from the included studies using a predefined Excel spreadsheet. Extracted data encompassed: (1) general study characteristics (e.g., author, publication year, country, study design, details of the surgical approaches employed, and follow‐up duration); as shown in (Table 1) (2) patient demographics and clinical characteristics (e.g., sample size per group, age, sex, indication for stoma creation, and prevalence of diabetes, smoking, and steroid use); as provided in (Table 2 [17, 18, 19, 20, 21, 22, 23, 24, 25, 26, 27]), and (3) clinical outcomes evaluated included short‐term outcomes, such as surgical site infection rate, urinary tract infection (UTI) rate, operative time, and length of hospital stay; as shown in (Table 3 [17, 18, 19, 20, 21, 22, 23, 24, 25, 26, 27]), and long‐term outcomes, including recurrence rate, reoperation rate, overall complication rate, and mortality rate; as provided in (Table 4 [17, 18, 19, 20, 21, 22, 23, 24, 25, 26, 27]). For the purpose of this analysis, the “recurrence rate” was defined as reported in the primary studies, which used heterogeneous detection methods including clinical examination, radiological imaging, or patient self‐report. While the recurrence rate reflects the detection of a recurrent hernia, the reoperation rate specifically refers to the need for subsequent surgical intervention.

**TABLE 2:** Patients' demographics.

Study	Study groups	Age (mean ± SD)	Males *n* (%)	Colostomy *n* (%)	Ileostomy *n* (%)	Urostomy *n* (%)	Diabetes *n* (%)	Steroid use (%)	Smoking *n* (%)
DeAsis et al. [17]	Laparoscopy sugar‐baker	62.4 ± 11.2	12 (48)	6 (28.6)	12 (57.1)	—	—	4 (16.0)	15 (60.0)
	Laparcopic keyhole	66.2 ± 11.6	11 (61.1)	8 (44.4)	10 (55.6)	—	—	6 (33.3)	7 (38.9)
	Open	63.8 ± 15.5	2 (33.3)	1 (16.7)	5 (83.3)	—	—	1 (16.7)	2 (33.3)
Dewulf et al. [18]	Laparoscopic	75.6 ± 4.02	3 (60)	—	—	5 (100)	0 (0)	—	1 (20)
	Robotic‐assisted	75.5 ± 5.38	7 (70)	—	—	10 (100)	2 (20)	—	0 (0)
Halabi et al. [19]	Laparoscopic	63 ± 15.55	88 (39.64)	—	—	—	35 (15.76)	7 (3.15)	34 (15.32)
	Open	63 ± 14	868 (44.63)	—	—	—	377 (19.38)	114 (5.86)	384 (19.74)
Helgstrand et al. [20]	Laparoscopic	65 ± 10.5	57 (48.3)	89 (75.4)	29 (24.6)	—	—	—	—
	Open	71 ± 16.5	23 (41.1)	32 (57.1)	24 (42.9)	—	—	—	—
Javaid et al. [21]	Laparoscopy and open‐group	64.51 ± 10.32	29 (61.7)	29 (61.7)	18 (38.3)	—	—	—	—
Keller et al. [22]	Open	58 ± 15	15 (48)	—	—	—	11 (36)		3 (10)
	Laproscopic	63 ± 11	11 (36)	—	—	—	6 (19)		6 (19)
Kohler et al. [23]	Laparoscopy and open‐group	68.4 ± 26.1	73 (54)	120 (88.9)	10 (7.4)	5 (3.7)	17 (12.6)	3 (2.2)	—
Lambrecht [24]	Laparoscopy and robotic‐assisted group	64.5 ± 9.6	10 (66.7)	10 (66.7)	3 (20)	—	2 (13.3)	—	3 (20)
McLemore et al. [25]	Open	64 ± 15	14 (46.67)	8 (26.67)	14 (46.67)	8 (26.67)	—	—	—
	Laproscopic	66 ± 12	12 (63.16)	5 (26.32)	5 (26.32)	9 (47.37)	—	—	—
Reali et al. [26]	Open	61.9 ± 15.92	19 (42.22)	—	—	—	7 (17.07)	3 (7.32)	—
	Laproscopic	60.27 ± 12.49	8 (44.44)	—	—	—	4 (22.22)	0 (0)	—
Ripoche et al. [27]	Laparoscopy and open group	72.3 ± 11.9	445 (63.5)	486 (62)	113 (15)	180 (23)	92 (12)	44 (5.8)	—

**TABLE 3:** Short‐term outcomes.

Study	Surgical site infection				UTI				Operative time						Length of hospital stay					
	Laparoscopic		Open		Laparoscopic		Open		Laparoscopic			Open			Laparoscopic			Open		
	*n*	Total	*n*	Total	*n*	Total	*n*	Total	Mean	SD	Total	Mean	SD	Total	Mean	SD	Total	Mean	SD	Total
DeAsis et al. [17]	2	25	1	6	1	25	0	6												
	3	18			0	18														
	5	43	1	6	1	43	0	6												
Dewulf et al. [18]									150	56	5	251	106	10	7.8	4	5	6	7	10
Halabi et al. [19]	194	1945	7	222	100	1945	138	80	222	153	64	1945	4	3	222	8	4	1945		
Helgstrand et al. [20]															3	8	118	7	15	56
Javaid et al. [21]																				
Keller et al. [22]	4	30	18	31					178	55	30	254	58	31	4	3	30	8	6	31
Kohler et al. [23]																				
Lambrecht [24]									126	36	6	177	89	9						
McLemore et al. [25]	2	19	2	39	2	19	4	39	208	58	19	146	111	39	6	3	19	5	4	39
Reali et al. [26]									50		18	62		45	3	2	18	7	6	45
Ripoche et al. [27]																				

**TABLE 4:** Long‐term outcomes.

Study	Recurrence				Re‐operation				Total complications				Mortality			
	Laparoscopic		Open		Laparoscopic		Open		Laparoscopic		Open		Laparoscopic		Open	
	Number	Total	*n*	Total	*n*	Total	*n*	Total	*n*	Total	*n*	Total	*n*	Total	*n*	Total
DeAsis et al. [17]	4	25	2	6	1	25	1	6	10	25	5	6	0	25	1	6
	11	18			1	18			15	18			0	18		
	15	43	2	6	2	43	1	6	25	43	5	6	0	43	1	6
Dewulf et al. [18]	0	5	1	10	0	5	1	10	3	5	4	10				
					0	5	3	10	0	5	2	10	0	5	0	10
Halabi et al. [19]								26	222	526	1945	1	222	31	1945	
Helgstrand et al. [20]					10	118	13	56					2	118	9	56
Javaid et al. [21]	7	29	7	18									0	29	0	18
Keller et al. [22]	6	30	20	31	1	30	3	31								
Kohler et al. [23]	13	63	31	67	8	63	20	67	11	63	13	67				
Lambrecht [24]	1	6	0	9	2	6	0	9								
McLemore et al. [25]					1	19	0	39					0	19	1	39
Reali et al. [26]	8	18	15	45					4	18	25	45				
Ripoche et al. [27]	3	9	28	63												

### 2.5 Quality Assessment

Two independent reviewers assessed the methodological quality of the included studies. The Newcastle–Ottawa Scale (NOS) [28] was used in cohort studies as provided in Table 5 [17, 18, 19, 20, 21, 22, 23, 24, 25, 26, 27]. The NOS employs a star‐rating system across three domains: selection, comparability, and outcome assessment. Disagreements between reviewers were resolved through discussion or by consulting a senior reviewer.

**TABLE 5:** Newcastle–Ottawa scale (NOS) quality assessment of non‐randomized studies.

	Cohort studies									
	Selection				Comparability	Outcome			Quality score	Quality
Study title	Representativeness of the exposed cohort	Selection of the non‐exposed cohort	Ascertainment of exposure	Demonstration that outcome of interest was not present at start of study	Comparability of cohorts on the basis of the design or analysis	Assessment of outcome	Was follow‐up long enough for outcomes to occur	Adequacy of follow up of cohorts		
Keller et al. [22]	♣	♣	♣	♣	♣				6	Fair
Kohler et al. [23]	♣		♣		♣		♣	♣	6	Fair
Lambrecht [24]	♣	♣	♣	♣		♣		♣	7	Poor
McLemore et al. [25]	♣	♣	♣	♣	♣			♣	7	Good
Reali et al. [26]	♣	♣	♣	♣	♣	♣		♣	8	Good
Ripoche et al. [27]	♠	♣	♣	♣	♣		♣	♣	8	Good
DeAsis et al. [17]	♣	♣	♣	♣	♣♣	♣	♣	♣	9	Good
Dewulf et al. [18]		♣	♣	♣		♣	♣	♣	6	Poor
Halabi et al. [19]	♣	♣	♣	♣	♣♣	♣		♣	8	Good
Helgstrand et al. [20]		♣	♣	♣	♣	♣	♣	♣	7	Good
Javaid et al. [21]		♣	♣	♣	♣		♣	♣	6	Fair

**FIGURE 3:**
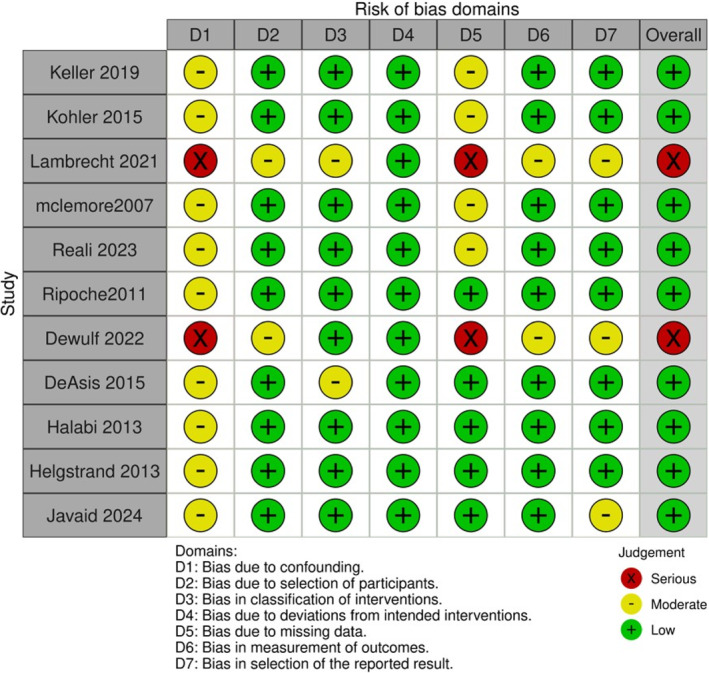
Risk of bias assessment according to ROBINS‐I domains.

In addition, domain‐specific risk of bias (ROB) was independently evaluated by two reviewers across all included studies. This assessment followed structured criteria focusing on selection bias, performance bias, detection bias, attrition bias, and reporting bias. Each domain was rated as having low, unclear, or high risk of bias based on available information. Discrepancies were resolved through discussion or with input from a senior reviewer. The summarized risk of bias across domains is presented in (Figure 3).

GRADE approach was applied to investigate the overall certainty in the evidence generated by the outcomes of our meta‐analysis. The GRADE approach yields an assessment of the quality of a body of evidence in one of four categories for each outcome: high, moderate, low, or very low [29] as shown in (Table 6).

**TABLE 6:** GRADE summary of evidence assessment.

Certainty assessment							No of patients		Effect		Certainty
No of studies	Study design	Risk of bias	Inconsistency	Indirectness	Imprecision	Other considerations	Laparoscope	Other approach	Relative (95% CI)	Absolute (95% CI)	
Length of hospital stay											
5	Non‐randomized studies	Serious^a^	Not serious	Not serious	Not serious	None	388	2077	RR −4.00 (−4.41 to −3.59)	0 fewer per 1000 (from 0 fewer to 0 fewer)	⨁ very low
Operative time											
5	Non‐randomized studies	Serious^b^	Very serious^c^	Not serious	Serious^d^	None	282	2034	MD 114.66 (95.71 to 133.62)	MD 114.66 higher (95.71 higher to 133.62 higher)	⨁ very low
Surgical site infection											
4	Non‐randomized studies	Not serious	Serious^e^	Not serious	Not serious	None	17/314 (5.4%)	281/2021 (13.9%)	RR 0.37 (0.15 to 0.90)	88 fewer per 1000 (from 118 fewer to 14 fewer)	⨁ very low
Urinary tract infection (UTI)											
3	Non‐randomized studies	Not serious	Not serious	Not serious	Not serious	None	10/284 (3.5%)	104/1990 (5.2%)	RR 0.65 (0.33 to 1.28)	18 fewer per 1000 (from 35 fewer to 15 more)	⨁⨁ low
Recurrence											
8	Non‐randomized studies	Serious^f^	Not serious	Not serious	Not serious	None	45/185 (24.3%)	89/204 (43.6%)	RR 0.52 (0.37 to 0.73)	209 fewer per 1000 (from 275 fewer to 118 fewer)	⨁ very low
Reoperation											
7	Non‐randomized studies	Serious^g^	Not serious	Not serious	Not serious	None	24/284 (8.5%)	41/218 (18.8%)	RR 0.48 (0.30 to 0.75)	98 fewer per 1000 (from 132 fewer to 47 fewer)	⨁ very low
Overall complication											
5	Non‐randomized studies	Serious^h^	Serious^i^	Not serious	Not serious	None	66/346 (19.1%)	569/2063 (27.6%)	RR 0.57 (0.39 to 0.84)	119 fewer per 1000 (from 168 fewer to 44 fewer)	⨁ very low
Mortality versus open techniques											
5	Non‐randomized studies	Not serious	Not serious	Not serious	Not serious	None	3/431 (0.7%)	42/2064 (2.0%)	RR 0.18 (0.06 to 0.49)	17 fewer per 1000 (from 19 fewer to 10 fewer)	⨁⨁ low
Mortality versus other techniques											
6	Non‐randomized studies	Serious^j^	Not serious	Not serious	Not serious	None	3/436 (0.7%)	42/2074 (2.0%)	RR 0.18 (0.06 to 0.49)	17 fewer per 1000 (from 19 fewer to 10 fewer)	⨁ very low

Abbreviations: CI, confidence interval; MD, mean difference; RR, risk ratio.

^a^ The proportion of information from studies at moderate risk of bias (5/5) is sufficient to affect the interpretation of results.

^b^ The proportion of information from studies at high risk of bias (2\5) is sufficient to affect the interpretation of results.

^c^Represent highly significant heterogeneity *I* ^2^ = 88% (*p* = 0.00001).

^d^ Wide confidence interval (−76.90 to 14.27).

^e^ Represent moderate heterogeneity *I* ^2^ = 51% (*p* = 0.11).

^f^ The proportion of information from studies at high risk of bias (2\8) is sufficient to affect the interpretation of results.

^g^ The proportion of information from studies at high risk of bias (2\7) is sufficient to affect the interpretation of results.

^h^ The proportion of information from studies at high risk of bias (1\5) is sufficient to affect the interpretation of results.

^i^ Might represent moderate heterogeneity, *I* ^2^: 49% (*p* = 0.12).

^j^ The proportion of information from studies at high risk of bias (1\6) is sufficient to affect the interpretation of results.

### 2.6 Statistical Analysis

Meta‐analyses were performed using Review Manager (RevMan) software version 5.4. Pooled risk ratios (RRs) with 95% confidence intervals (CIs) were calculated for dichotomous outcomes (e.g., recurrence rate, reoperation rate, overall complication rate, mortality rate, surgical site infection rate, and UTI rate). Mean differences (MDs) with 95% CIs were calculated for the continuous outcomes (e.g., operative time and length of hospital stay). Heterogeneity was assessed using the *p*‐value, with a *p*‐value < 0.1 considered indicative of substantial heterogeneity, following the guidelines outlined in the Cochrane handbook for systematic reviews of interventions [30]. A random‐effects model was applied for analyses with substantial heterogeneity; otherwise, a fixed‐effects model was used. In cases of high statistical heterogeneity, a leave‐one‐out sensitivity analysis was conducted to identify potential outlier studies and to assess the stability of the pooled effect estimate. Subgroup analyses were performed based on the comparative technique, focusing on open repair versus minimally invasive approaches, specifically the laparoscopic technique and, to a lesser extent, the robotic‐assisted technique.

## 3 Results

In total, detailed stoma‐type data were available for 13 groups across 7 studies, covering 1254 patients. Among these, 794 patients (63.3%) had a colostomy, 243 patients (19.4%) had an ileostomy, and 217 patients (17.3%) had a urostomy. For example, Helgstrand et al. reported 89 colostomies and 29 ileostomies in the laparoscopic group, and 32 colostomies and 24 ileostomies in the open group. Kohler et al. reported 120 colostomies (88.9%), 10 ileostomies (7.4%), and 5 urostomies (3.7%). Ripoche et al. included 486 colostomies (62%), 113 ileostomies (15%), and 180 urostomies (23%). Dewulf et al. reported exclusively urostomies with 5 and 10 patients in the laparoscopic and robotic‐assisted groups, respectively. DeAsis et al. detailed the proportions of colostomies and ileostomies in both laparoscopic and open subgroups, with colostomies ranging from 16.7% to 44.4% and ileostomies from 55.6% to 83.3%. McLemore et al. reported a more evenly distributed mix in both groups. In most cases, colostomies were placed in the left lower quadrant, while ileostomies and urostomies were primarily located in the right lower quadrant, in line with standard surgical practice, as per Table 2. In addition to stoma characteristics, we analyzed key patient comorbidities that could influence surgical outcomes. The prevalence of diabetes mellitus ranged from 0% to 36% across the groups where it was reported, with the largest study by Halabi et al. showing comparable rates between laparoscopic (15.8%) and open (19.4%) cohorts. Smoking and steroid use were also documented, though rates varied considerably (Table 2).

Risk of bias was generally low to moderate across studies, with most showing low risk in detection and reporting domains, and moderate concerns in selection and attrition bias (Figure 3). Overall, the methodological quality was acceptable.

The GRADE assessment indicated very low to low certainty of evidence for most outcomes, mainly because of retrospective study designs, heterogeneity, and imprecision (Table 6). Operative time, recurrence, and complications were rated very low while UTI and mortality showed low certainty.

**FIGURE 4:**
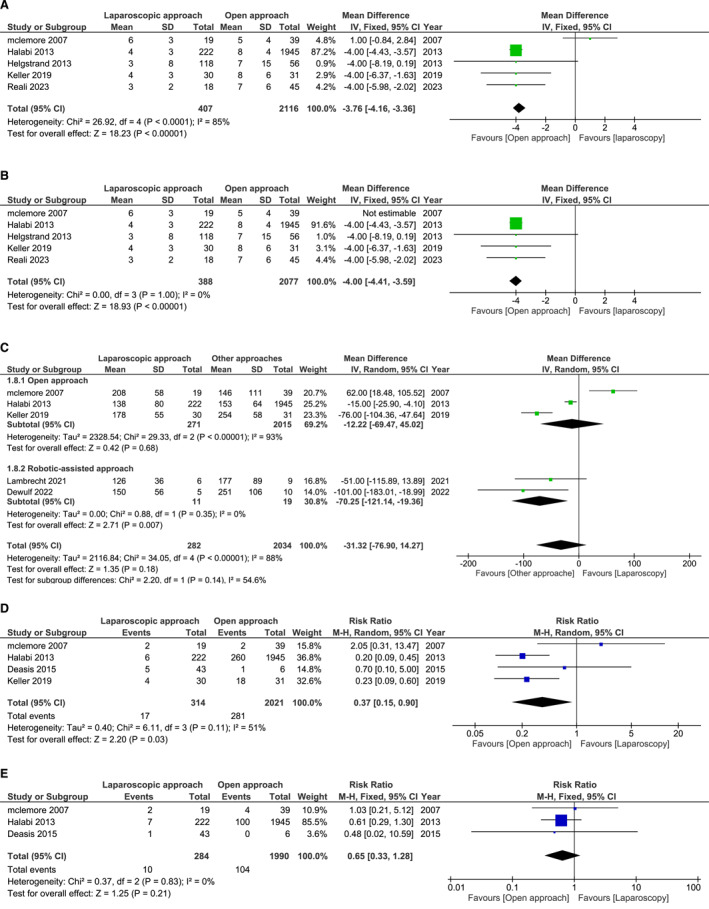
Short‐term outcomes Forest plots.

The analysis of our forest plots in Figure 4 highlights several significant findings regarding short‐term outcomes in parastomal hernia repair, comparing laparoscopic‐assisted and open techniques.

### 3.1 Length of Hospital Stay

Forest plot‐A shows that the laparoscopic approach significantly reduces hospital stay compared to the open technique, with a mean reduction of up to 4 days (*p* < 0.00001, RR = 0.45). This reduction likely reflects the laparoscopic approach's role in promoting quicker recovery and reducing hospital resource utilization.

### 3.2 Operative Time

Forest plot‐B compares operative times across surgical approaches. Minimally invasive techniques—particularly robotic‐assisted surgery—showed shorter operative times than open surgery. Despite being based on limited data (19 patients from two studies [18, 24]), robotic‐assisted repair reduced operative time by approximately 70 min (RR = 0.60, *p* = 0.007). Given the small sample size, this observation should be interpreted cautiously and not considered conclusive.

### 3.3 Surgical Site Infection

Forest plot‐C assesses postoperative surgical site infection rates, with laparoscopic surgery showing a notably significantly lower incidence than open surgery (RR = 0.37, *p* = 0.02). Smaller incisions in minimally invasive procedures likely contribute to reduced infection risks, leading to improved recovery and fewer additional postoperative treatments.

### 3.4 Urinary Tract Infection

Forest plot‐D compares urinary tract infection (UTI) rates, finding no statistically significant difference between laparoscopic and open techniques (RR = 1.05, *p* = 0.18). The similar UTI rates suggest that catheterization needs across both approaches do not significantly impact UTI incidence.

**FIGURE 5:**
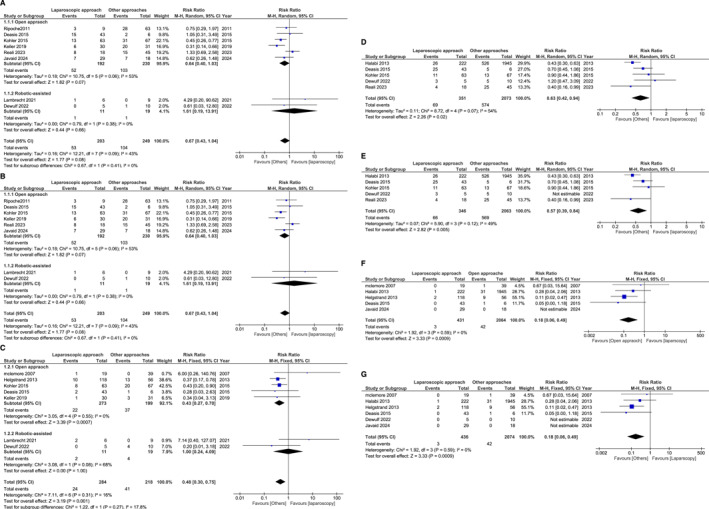
Long‐term outcomes Forest plots.

The analysis of long‐term outcomes in Figure 5 provides key findings on recurrence, reoperation, overall complications, and mortality, comparing laparoscopic‐assisted and open approaches.

### 3.5 Recurrence Rate

Forest plot‐A assesses recurrence rates before the leave‐one‐out test, with Forest plot‐B showing results after. The laparoscopic approach has a comparable recurrence rate to the open method with no statistically significant difference (*p* = 0.21, RR = 1.12). This consistency across tests suggests that recurrence rates are not significantly influenced by the surgical approach.

### 3.6 Reoperation

Forest plot‐C shows that the laparoscopic approach has significantly lower reoperation rates than the open method (RR = 0.43, *p* = 0.0007). This reduction supports the durability of laparoscopic repair in preventing complications that necessitate additional surgery, thus contributing to greater patient satisfaction and reduced long‐term healthcare costs.

### 3.7 Overall Complications

Forest plot‐D presents overall complication rates before the leave‐one‐out test, with Forest plot‐E showing results after. The laparoscopic approach results in significantly fewer complications compared to the open technique, with risk ratios between 0.37 and 0.63 and *p*‐values from 0.02 to 0.03. This reduction in complications remains significant after sensitivity testing, confirming that minimally invasive surgery benefits long‐term patient outcomes by reducing morbidity.

### 3.8 Mortality Rate

Forest plot‐F compares mortality between laparoscopic and open methods while Forest plot‐G evaluates mortality rates between laparoscopic and other techniques. Laparoscopic surgery was associated with significantly lower mortality (RR = 0.18, *p* = 0.0009), suggesting a survival benefit likely linked to reduced complication rates.

Overall, these results demonstrate that minimally invasive approaches offer benefits over traditional open surgery in the repair of parastomal hernias. Reduced hospital stays, lower complication rates, fewer reoperations, and lower mortality rates underscore the advantages of minimally invasive techniques in achieving superior patient outcomes.

## 4 Discussion

This systematic review and meta‐analysis was conducted to comprehensively evaluate and compare the clinical outcomes of open versus minimally invasive techniques in parastomal hernia repair, focusing on key parameters such as operative time, hospital stay, recurrence rates, postoperative complications, and overall patient outcomes.

Our findings reinforce the growing body of evidence supporting the benefits of minimally invasive surgery, in reducing morbidity, shortening hospitalization, and improving postoperative recovery [31].

These advantages are largely attributable to the reduced tissue trauma associated with minimally invasive approaches, which facilitate faster healing and fewer complications. Previous studies have similarly demonstrated that laparoscopic repairs are often associated with shorter hospital stays compared to open surgery [3, 5, 14]. For example, Ye et al. and Olavarria et al. linked reduced length of stay to the minimally invasive nature of these techniques [13, 32], although institutional protocols and patient selection may also influence this outcome [13].

To a lesser extent in our meta‐analysis, the robotic‐assisted surgery demonstrated significant reductions in operative times over open surgery, averaging a decrease of approximately 70 min (*p* = 0.007). This is consistent with Lai et al., who emphasized the role of robotic systems in enhancing precision and efficiency [31] and saving time during surgical procedures. However, it is important to note that the robotic‐assisted procedures included only 19 patients within two of our included studies [18, 24], which limits the generalizability of this finding. Given this small sample size, the observed reductions in operative time may not accurately reflect real‐world surgical performance, where robotic procedures often involve additional setup time and a learning curve [33]. Additionally, robotic‐assisted procedures still generally require 30–60 min more than laparoscopic approaches due to setup complexity and the steep learning curve, though time savings increase with surgeon experience [31, 32]. Despite improved efficiency with experience, the setup phase remains a logistical and cost burden for many institutions [34].

A wide variety of surgical techniques were employed for parastomal hernia repair across the included studies. The most commonly used approaches were the Sugarbaker and keyhole techniques, both of which are standard in minimally invasive hernia repair [17, 18, 20, 22, 24, 26]. However, other techniques such as sublay, onlay, primary suture repair, modified Sugarbaker, and TAR (transversus abdominis release) were also reported, particularly in open or robotic‐assisted procedures [18, 19, 21].

This variation reflects the absence of a universally adopted standard for parastomal hernia repair and underscores the complexity of comparing outcomes across different studies. Importantly, our subgroup analysis did not reveal statistically significant differences between these surgical techniques, limiting the ability to draw firm conclusions about the superiority of one method over another.

A recent meta‐analysis evaluating open surgical techniques for parastomal hernia repair—comparing direct repair to stoma relocation—analyzed outcomes in approximately 4000 patients, with around 2000 in each group. The findings indicated that direct repair may offer advantages in terms of shorter operative time and hospital stay while stoma relocation was significantly associated with a lower rate of reoperations. Overall, both approaches demonstrated comparable safety and efficacy, with no significant differences in recurrence, complication rates, or mortality. Therefore, neither technique showed definitive superiority [35].

Laparoscopic techniques are associated with lower mortality and fewer complications than open surgery, supporting Zhen Shi, comparative analysis findings on the survival advantage of minimally invasive methods [34]. Patient‐specific factors, including age and surgery urgency, also affect survival outcomes, highlighting the need for individualized treatment plans [13, 32].

Our study further indicates that laparoscopic methods reduce complication rates, particularly surgical site infections, with risk ratios between 0.37 and 0.63. This reduction is consistent with Kulkarni et al. [36] who noted fewer wound complications due to the reduced tissue trauma in laparoscopic surgery. Wang et al. observed that this advantage is more pronounced in elective cases compared to emergencies, where the benefits may be less significant [37].

Regarding urinary tract infections (UTIs), no significant differences were observed between laparoscopic and open methods. Li et al. reported similar UTI rates across methods, likely because of standardized perioperative care protocols [38].

Our findings reinforce the benefits of laparoscopic techniques, including shorter hospital stays, reduced operative times, decreased mortality, and fewer complications, supporting the shift toward minimally invasive surgery to improve patient outcomes [39]. However, whether robotic‐assisted repair consistently outperforms laparoscopic methods remains controversial [40, 41, 42]. A broader analysis is needed to assess case‐specific factors, particularly when operative times and costs are considered [32, 43, 44].

Robotic surgery generally costs 20%–30% more than laparoscopic repair due to increased operating room time and robotic equipment costs [45, 46]. Some studies argue that robotic repairs might reduce long‐term expenses by lowering complication rates, but evidence of this potential saving is mixed [9, 13, 39, 46]. This cost differential is particularly relevant for routine cases where laparoscopic surgery achieves similar outcomes at lower costs, making it the more economical option for many institutions [8, 38].

Importantly, although laparoscopic approaches offer superior short‐term outcomes, our analysis confirms that recurrence rates—a key long‐term parameter—do not significantly differ between laparoscopic and open repair. What probably matters more in the long‐term setting is the heterogeneity of surgical techniques, patient selection, and variability in follow‐up durations, which may dilute the measurable impact of the surgical approach itself [38].

As previously discussed, the choice between open and minimally invasive techniques does not appear to significantly influence hernia recurrence rates. However, laparoscopic repair was associated with a significantly lower reoperation rate (Risk ratio = 0.43), which may reflect improved intraoperative visualization and mesh positioning, resulting in more durable repairs and better management of postoperative complications. This suggests that, although recurrence may occur at similar rates, its clinical consequences may be mitigated more effectively with minimally invasive approaches. In line with this interpretation, Fry et al. found that disease characteristics influence recurrence rates more than the surgical technique itself [47].

Accurate detection of recurrence remains essential for evaluating long‐term outcomes. Although both CT and MRI are widely used for postoperative imaging, a recent meta‐analysis concluded that CT is superior to MRI in detecting hernia recurrence, particularly in identifying acute complications. In contrast, MRI is more effective for mesh visualization and soft‐tissue assessment. These findings underscore the importance of selecting the appropriate imaging modality based on clinical context to optimize postoperative monitoring and care [48], especially for patients with parastomal hernia.

Whereas minimally invasive methods reduce short‐term complications, they may not inherently lower recurrence risks [49, 50]. Robotic‐assisted techniques, with their potential for precise suturing and mesh placement, may help reduce recurrence in select cases, though further studies with extended follow‐up are needed [51].

This meta‐analysis has several limitations that warrant consideration. First, there was notable heterogeneity in follow‐up durations among the included studies, ranging from 12 to 285 months. Such variability may have influenced the reporting and comparability of long‐term outcomes, particularly recurrence and reoperation rates. Although we attempted to interpret these outcomes within the context of each study's follow‐up period, this inconsistency limits the strength of pooled long‐term conclusions.

The heterogeneity in surgical technique represents a major limitation of our meta‐analysis. Differences in mesh placement, fixation methods, surgeon experience, and patient selection may have influenced both perioperative and long‐term outcomes. This variability introduces potential bias and complicates direct comparisons across studies, possibly diluting the observed advantages of minimally invasive approaches. For example, the large retrospective study by Halabi et al. [19] included 2167 patients—222 undergoing laparoscopic repair and 1945 open repairs—without providing granular data on technique standardization or patient stratification, further contributing to methodological heterogeneity. Additionally, follow‐up durations across studies varied considerably, ranging from 12 months in Dewulf et al. [18] to 285 months in Reali et al. [26], reflecting another layer of heterogeneity that may have impacted the comparability and interpretation of long‐term outcomes. Future studies should aim to standardize surgical techniques or provide detailed, stratified outcome data to enhance comparability and precision.

The overall quality of evidence supporting our findings is limited, as indicated by the GRADE assessment. This reflects the predominance of retrospective designs, small sample sizes, and the heterogeneity of surgical techniques and follow‐up durations. Despite these constraints, our meta‐analysis offers a comprehensive synthesis of the current evidence and reveals important trends in clinical outcomes that remain valuable for guiding surgical decision‐making. However, these findings should be interpreted with caution. Whereas a leave‐one‐out sensitivity analysis might further clarify the impact of individual studies, we refrained from this approach in some parameters due to its resource demands and the overall consistency of our pooled estimates.

A further limitation lies in the small number of patients undergoing robotic‐assisted repair, which restricts our ability to draw firm conclusions about its comparative effectiveness. Combined with the low certainty of evidence and cost considerations, this limits the generalizability of our findings. To advance the field, future research must prioritize large‐scale prospective randomized trials—particularly those comparing robotic and laparoscopic approaches—with standardized techniques, longer follow‐up, and an emphasis on patient‐reported outcomes and cost‐effectiveness.

## 5 Conclusion

This meta‐analysis highlights the clear advantages of minimally invasive approaches—particularly laparoscopic surgery—for parastomal hernia repair including shorter hospital stays, fewer complications, lower mortality, and reduced reoperation rates. However, recurrence rates remained similar between open and laparoscopic techniques, suggesting that the long‐term outcome may be more influenced by the heterogeneity of surgical techniques and follow‐up durations than by the surgical approach itself. Therefore, a minimally invasive approach should be preferred whenever feasible, particularly for its short‐term benefits while acknowledging the need for standardized operative strategies to improve long‐term results.

Given the limited number of cases, the effectiveness of robotic‐assisted repair could not be conclusively assessed; however, preliminary evidence suggests it may enhance operative efficiency and surgical precision, especially in complex or recurrent cases.

## Author Contributions

**Ahmed Abdelsamad:** conceptualization, methodology, data curation, supervision, resources, formal analysis, project administration, validation, visualization, investigation, writing – original draft, writing – review and editing, software. **Mohammed Khaled Mohammed:** investigation, writing – original draft, writing – review and editing, visualization, validation. **Mohamed Badr Almoshantaf:** validation, visualization. **Aya Alrawi:** project administration, formal analysis, software. **Ziad A. Fadl:** resources, data curation, software. **Ziad Tarek:** methodology, visualization, project administration, resources. **Nada Osama Aboelmajd:** formal analysis, software, project administration, investigation. **Torsten Herzog:** supervision, writing – review and editing. **Florian Gebauer:** resources, supervision, data curation, project administration, visualization, validation, writing – review and editing. **Nada K. Abdelsattar:** methodology, software, investigation, data curation, resources, visualization. **Taha Abd‐Elsalam Ashraf Taha:** investigation, methodology, software, data curation, supervision, resources, formal analysis, visualization, writing – review and editing.

## Conflicts of Interest

The authors declare no conflicts of interest.

## Supporting information

Supporting Information S1

## Data Availability

All original data are available upon reasonable request to the corresponding authors.
