# Supplementary material for: Minimally Invasive Versus Open Parastomal Hernia Repair: A Comprehensive Systematic Review and Meta‐Analysis
Source: World J Surg. 2025 Jul 25;49(9):2382–98. doi: 10.1002/wjs.70013 (PMC12435612; doi:10.1002/wjs.70013)
Supplement: Supplementary file 1 — Supporting Information S1 [file WJS-49-2382-s001.docx]

# Supplementary Appendix 1 – Full Search Strategy

This appendix provides the complete search strategy used in the systematic review across four major databases: PubMed, Web of Science (WOS), Scopus, and Cochrane Library. The search was conducted up to October 5, 2024.

## Database: PubMed

Date of last search: October 5, 2024

(("Parastomal Hernia"[Mesh] OR "parastomal hernia" OR "parastomal hernias" OR "hernia near stoma" OR "stoma-related hernia") AND
("Hernia, Abdominal"[Mesh] OR "Enterocele" OR "Herniation" OR "Herniae") AND
("Surgical Procedures, Operative"[Mesh] OR "repair" OR "surgical repair") AND
("Minimally Invasive Surgical Procedures"[Mesh] OR "laparoscopic" OR "laparoscopy" OR "robotic-assisted" OR "robotic" OR "MIS") AND
("Open surgery" OR "open repair"))

## Database: Web of Science (WOS)

Date of last search: October 5, 2024

TS = (parastomal hernia OR herniae OR herniation OR parastomal hernias OR enterocele) AND
TS = (laparoscopy OR laparoscopic OR robotic-assisted OR minimally invasive surgery OR MIS OR robotic) AND
TS = (open repair OR surgical treatment OR operative outcomes)

## Database: Scopus

Date of last search: October 5, 2024

TITLE-ABS-KEY (“parastomal hernia” OR “herniae” OR “hernia repair” OR “enterocoele”) AND
TITLE-ABS-KEY (“laparoscopy” OR “robotic surgery” OR “robotic-assisted” OR “minimally invasive” OR “MIS”) AND
TITLE-ABS-KEY (“open repair” OR “surgical outcome” OR “operative time” OR “postoperative complications”)

## Database: Cochrane Library

Date of last search: October 5, 2024

(parastomal hernia OR herniation OR parastomal hernias) in Title Abstract Keyword AND
(laparoscopic OR robotic OR minimally invasive OR MIS) in Title Abstract Keyword AND
(open repair OR operative outcome OR surgical technique) in Title Abstract Keyword

## Filters Applied

- Language: English
- Population: Human
- Age: Adult (18+)
- Study types: Clinical trials, cohort studies, comparative studies
